# Treatment of UTIs Due to *Klebsiella pneumoniae* Carbapenemase-Producers: How to Use New Antibiotic Drugs? A Narrative Review

**DOI:** 10.3390/antibiotics10111332

**Published:** 2021-11-01

**Authors:** Caroline Chapelle, Benjamin Gaborit, Raphaëlle Dumont, Aurélien Dinh, Maxime Vallée

**Affiliations:** 1 Department of Urology, Poitiers University Hospital, 2 Rue de la Milétrie, 86021 Poitiers, France; chapelle.caroline07@gmail.com (C.C.); raphdum@hotmail.fr (R.D.); 2 Department of Infectious Diseases, University Hospital of Nantes and CIC 1413, INSERM, 44093 Nantes, France; Benjamin.GABORIT@chu-nantes.fr; 3 Department of Infectious Disease, University Hospital R. Poincaré, Garches, APHP, Paris Saclay University, 92380 Garches, France; aurelien.dinh@aphp.fr; 4 INSERM U1070, “Pharmacologie des Anti-Infectieux”, UFR Médecine-Pharmacie, Université de Poitiers, Pôle Biologie Santé, 1 rue Georges Bonnet, Bâtiment B36 TSA 51106, CEDEX 9, 86073 Poitiers, France

**Keywords:** UTI, KPC, cefiderocol, meropenem-vaborbactam, ceftazidim-avibactam, imipenem-relebactam, antibiotic treatment, PK/PD

## Abstract

Background: *K. pneumoniae* is one of the bacteria most frequently causing health care-associated urinary tract infections, and increasingly incriminating *Klebsiella pneumoniae* carbapenemase producers (KPCp). Most infections caused by KPCp are nosocomial and might cause serious issues, even leading to death in half of the reported cases. Our aim was to identify the best strategy, based on available scientific data, for the use of new antibiotic treatments to manage KPCp UTIs. Methods: this narrative review of the literature was performed according to the criteria of preferred reporting items for systematic review and meta-analyses statement (PRISMA) (2020). Results and Conclusions: KPCp-UTIs are a real challenge for physicians. While cefiderocol, meropenem-vaborbactam, ceftazidim-avibactam, and imipenem-relebactam represent a major step forward in the treatment of these UTIs, no guidelines are currently available, in view of choosing the most appropriate treatment, in each specific case.

## 1. Introduction

Due to an increased rate of antibiotic resistance, experts from the World Health Organization (WHO) have predicted that the incidence of morbidity due to infectious diseases will be similar in 2050 to what it was in the “pre-antibiotic era”. This prediction is being confirmed nowadays, as was demonstrated by Cassini in 2018 [1]. The dramatic explosion of bacterial resistance is even more obvious among enterobacterales and, as such, represents an important challenge for the future [1,2]. For instance, the WHO has made it a priority to develop new antibiotic treatments [3]. In 2014, the Global Report on Surveillance of antimicrobial resistance by the World Health Organization [4] showed that enterobacterales, especially *K. pneumoniae*, were common in hospitals, particularly in respiratory tract and urinary tract infections (UTI), in vulnerable patients and that they might very easily spread among departments, hospitals, and countries. The report highlighted alarming rates of carbapenem resistance, largely exceeding fifty percent, in certain patient groups, for whom there are few (or possibly no) other treatment options.

*K. pneumoniae* is one of the bacteria that cause healthcare-associated urinary tract infections and the increase of cases caused by *Klebsiella pneumoniae* carbapenemase-producers (KPCp) have been reported [5]. Most infections caused by KPCp are nosocomial and cause serious issues, even leading to death in half of the reported cases [6]. In the Ambler classification system, KPC belongs to the clade A of β-lactamases, which involve serine at their active site and hydrolyze numerous penicillins, cephalosporins, and carbapenems [7]. Compared to Clade B (NDM, VIM, IMP) and clade C (OXA-48), clade A KPC carbapenemase is the most frequent mechanism of acquired carbapenem resistance in *K. pneumoniae* [8]. Indeed, KPC is the one of the most prevalent carbapenemase producers worldwide and one of the most widely studied mechanisms of resistance [9].

Medical care of KPCp UTIs is difficult nowadays, due to a lack of rapid and reliable diagnostic tests to identify carbapenamase and because there are no specific antibiotics to treat them. This leads to the use of late drug combinations and increases the risk of widespread infections, due to drug-acquired resistance [10]. However, the availability of new antibiotic treatments, discovered in the last decade, represents a new hope for these patients. That said, the data on pharmacokinetics/pharmacodynamics (PK/PD) and dosage of these new antibiotics in urines may represent a highly challenging situation, especially when choosing the most appropriate treatment for very specific clinical situations.

Our aim was to identify the best strategy, based on available scientific data, for application of new antibiotic treatments for management of KPCp UTIs.

## 2. Materials and Methods

This narrative review of the literature was performed according to the criteria of preferred reporting items for systematic review and meta-analyses statement (PRISMA) (2020) [11].

### 2.1. Eligibility Criteria

Criteria for the studies to be considered were all studies investigating the treatment of KPCp, associated with urinary tract infections in patients over the age of 18.

A cut-off date was not used to select the articles because only a very limited number of studies were available matching the criteria.

The studies selected in this review were then matched, according to the type of antibiotic treatment and medical strategy.

### 2.2. Search Strategy

After research on KPCp etiology, diagnostic, and treatment available, a review of the literature was performed in Medline library on 15 April 2021, using the following MeSH keywords and search algorithms: ((carbapenem-resistant *Klebsiella pneumoniae* OR KPC) AND (UTI OR urinary tract infection OR prostatitis OR pyelonephritis OR cystitis OR orchitis OR epididymitis) AND (antibiotic OR antibiotic treatment OR antibiotic therapy OR treatment OR cefiderocol OR vaborbactam OR avibactam OR relebactam OR eravacycline) NOT pediatric). Articles were screened for methodology, language (English/French), and pertinence to this review. An Internet search yielded additional references, including guidelines.

### 2.3. Study Selection and Data Extraction

**Figure 1:**
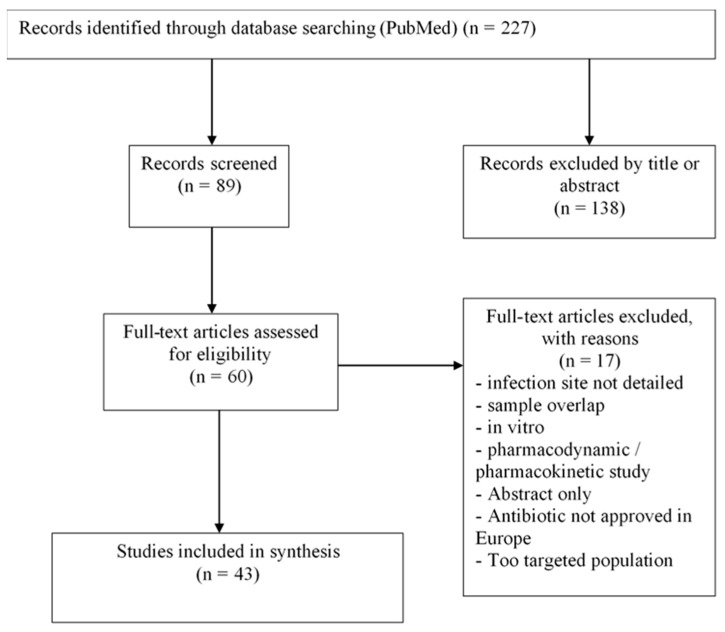
Flow-chart.

All articles were selected by CC, who was then audited by MV. All the studies collected were considered. The PRISMA flowchart is represented on Figure 1.

Only data reporting the application of antibiotic treatments for adult UTIs linked with KPCs were collected, as assessed in the eligibility criteria. Studies with unavailable abstracts were excluded immediately, as were basic research studies.

All studies using animal models or non-representative study population were excluded. Studies with languages other than English and French were excluded.

The first part of this systematic review consisted in defining infections caused by KPCp and finding its risk factors, clinical situations, and associated population. The second part of the review presents the different antibiotics available and their potential usage.

We conducted additional research to add posology, PK/PD data, and tips about every cited antibiotic. We did not include these studies in the flow chart.

## 3. Results

Forty-three studies were included in the synthesis.

### 3.1. KPCp Description and Population

The usual mechanism of carbapenem resistance worldwide is the production of *Klebsiella pneumoniae* carbapenemase enzymes, which are encoded by the blaKPC gene and have spread to numerous Gram-negative species [12]. This enzyme is able to hydrolyze carbapenem antibiotics and leads to resistances to the latest generations of antibiotics [13,14]. In the Ambler classification system, they belong to the clade A of β-lactamases, which integrates serine to their active site and hydrolyzes numerous penicillins, cephalosporins, and carbapenems [15].

They constitute the major transmissible genes found in enterobacterales, in this class [16]. They are mainly produced by *K. pneumoniae* but can also be found in *Salmonella enterica, E. coli, K. oxytoca*, and *Pseudomonas aeruginosa* too [17]. They have had a very quick propagation since 2000 from New-York [18], throughout the USA and South American countries, and in several European countries, mostly Mediterranean countries and Israel. Nowadays, KPCp represents the most frequent pathogen of carbapenemase-producing enterobacterales [19].

Numerous studies have reviewed the clinical characteristics and outcomes of patients infected with KPCp. They tend to be older, feebler, and have various complications like diabetes melitus and immune deficiency. These patients regularly attend to hospitals where they may undergo surgery or complex care, in the context of their different diseases [12,20]. KPCp is more often found in respiratory tract, urinary tract, and bacteremia and is greatly associated with prosthetic devices, such as indwelling urinary catheter and mechanical ventilation [21,22]. Some research has proven that urological tumors could be a risk factor for KPCp UTIs too [20]. Infections caused by KPCp are mostly linked to healthcare infections, a factor necessitating prevention.

The Table 1 and Table 2 summarize the main studies and antibiotics included in this analysis.

### 3.2. Effective Antibiotics

#### 3.2.1. CEFIDEROCOL (FETROJA^®^)

##### Antimicrobial Spectrum of Activity and Resistance

Cefiderocol is an injectable siderophore cephalosporin. It has intrinsic structural stability against a variety of Ambler class A, C and D β-lactamases, and even B by having activity against multidrug-resistant (MDR) Gram-negative bacilli. It has a safety and tolerance comparable to the rest of the cephalosporins. Cefiderocol appears to be a key antibiotic in countering the spread of infections caused by carbapenem-resistant and MDR Gram-negative bacilli. This includes extended-spectrum β-lactamase and carbapenemase-producing strains, as well as carbapenem-resistant strains without carbapenemase producing enzyme [23].

Its innovative mechanism of action is a combination of two characteristics: a siderophore-like cellular entry creating a high concentration of antibiotics at the locus of action and resistance to hydrolysis, caused by almost all β-lactamases, especially serine- and metallo-β-lactamases. This explains its superior antimicrobial activity, compared with carbapenems, β-lactam/β-lactamase combinations, and modern cephalosporins [24,25].

Recent studies like the CREDIBLE trial, which included more than 100 patients, compare cefiderocol with the best available treatment. It was a multicenter trial, involving 95 centers, randomized 2 on 1, with multiple indications, and more than 50% of severe patients. It showed that clinical and microbiological outcomes were comparable between the cefiderocol group and the group treated with the best available therapy, whatever the carbapenem-resistant pathogen or infection site [26,27].

##### Clinical Efficacy, Safety and Tolerability

A meta-analysis assessed the clinical efficacy and safety of cefiderocol in the treatment of acute bacterial infections, compared to other therapies, such as carbapenem. It shows no significant difference, in response to treatment rate at the end of cure, between cefiderocol and control groups (OR = 1.04, 95% CI 0.73–1.48; *I*^2^ = 0%).

All-cause mortality did not differ between the cefiderocol and comparators (14-day mortality, OR = 1.25, 95% CI 0.69–2.26; *I*^2^ = 0%; and 28-day mortality, OR = 1.12, 95% CI 0.69–1.82; *I*^2^ = 0%).

Moreover, Cefiderocol was shown to have a microbiological response, comparable to control groups at the end of the cure (OR = 1.44, 95% CI 0.84–2.47; *I*^2^ = 63%) [28].

The study highlights a risk of adverse events (AEs), comparable to comparators (best available therapy known or carbapenem) [28]. In a phase II trial, comparing cefiderocol versus imipenem-cilastatin in complicated urinary tract infection (cUTI), gastrointestinal disorders like diarrhoea (4%), and constipation (3%), were the most frequent AEs in the cefiderocol group [29]. Similarly, a phase III trial, comparing the safety and efficacy of cefiderocol in serious infections, caused by carbapenem-resistant Gram-negative bacteria, to the best available therapy, showed that the most frequently reported adverse events in the cefiderocol group were diarrhea (19%), fever (14%), and vomiting (13%) [26].

##### Posology

Due to their time-dependent PK/PD, all infusions have to be administered within 3 h to optimize efficacy. The suggested dosage of cefiderocol is 2 g administered every 8 h in patients 18 years of age and older, with an estimated glomerular filtration rate (eGFR) superior to 90 mL/min/1.73 m^2^. The treatment should last between five to ten days, in case of cUTI. When creatinine clearance is superior to 120 mL/min, a regimen of 2 g every 6 h can be used. The recommended dose of cefiderocol in renally impaired patients with eGFR between 30–60 mL/min/1.73 m^2^ is 1.5 g every 8 h, for 15–30 mL/min/1.73 m^2^, 1 g every 8 h, and for less than 15 mL/min/1.73 m^2^, 0.75 g every 12 h [30,31].

##### PK/PD

Urinary excretion ranged from 61.5% to 68.4% unchanged antibiotic product, regardless of the dosage but no specific data are available for prostatic diffusion [32].

#### 3.2.2. Meropenem/Vaborbactam (Vaborem)

##### Antimicrobial Spectrum of Activity and Resistance

Meropenem/vaborbactam was accepted in August 2017 by the FDA for the treatment of cUTI including AP [33]. Meropenem has a wide spectrum of activity against aerobic and anaerobic Gram-positive and Gram-negative organisms, including drug-resistant strains like ESBL- and AmpC-producing enterobacterales [34]. However, the production of β-lactamases, such as KPC serine carbapenemases leads to carbapenem resistance [35].

Vaborbactam (acid boronic) is a non-β-lactam, serine beta-lactamase inhibitor, which restores the activity of meropenem against enterobacterales producing Ambler clade A β-lactamases but is not effective on clade B and D [36].

##### Clinical Efficacy, Safety, and Tolerability

The usefulness and risks of M/V to treat cUTIs, due to known or suspected CRE, have been estimated in two Phase III trials: TANGO-I and TANGO-II [37,38]. High overall clinical success rates were recorded in the meropenem-vaborbactam group (98.4%) versus 94.0% in the piperacillin-tazobactam group (difference 4.5%, [95% CI: 0.7–9.1%]; *p* < 0.001 for non-inferiority; *p* = 0.01 for superiority).

At the end of the therapies, patients who received meropenem–vaborbactam more often completed the clinical cure (64.3 vs. 33.3%; *p* = 0.04), in comparison with patients who had received the best available therapy (BAT) [39]. This trial was prematurely stopped because of the superiority of M/V compared to BAT. This could be explained by the high proportion of severe adverse events in the BAT group probably, due to the use of colistin.

M/V shows a better safety profile compared to BAT and presents fewer adverse events (24.4% vs. 44%) [40]. These are described as mild to moderate and refer to: headache (3.8–21.6%), infusion site phlebitis (42–62.2%), nausea (19.5%), and diarrhea (14.6%) [41].

##### Posology

The suggested dosage of meropenem-vaborbactam is 2 g/2 g administered every 8 h in patients 18 years of age and older, with an estimated glomerular filtration rate (eGFR) superior to 50 mL/min/1.73 m^2^ [42], for a duration of five to ten days. The mentioned dose of meropenem-vaborbactam, in renally impaired patients with an eGFR between 30–49 mL/min/1.73 m^2^ is 2 g (meropenem 1 g and vaborbactam 1 g) every 8 h, for 15–29 mL/min/1.73 m^2^ is 2 g every 12 h, and for less than 15 mL/min/1.73 m^2^ is 1 g (meropenem 0.5 g and vaborbactam 0.5 g) every 12 h. All infusions should be administered over 3 h [40]. Dose adjustments are probably not necessary in patients with hepatic impairment, as both substances undergo minimal or no hepatic metabolism [41].

##### PK/PD

The urinary excretion of Meropenem and Vaborbactam a ranged from 40 to 60% and 75 to 95%, respectively. No specific data are available for prostatic diffusion [43].

#### 3.2.3. Ceftazidime–Avibactam (Zavicefta^®^)

##### Antimicrobial Spectrum of Activity and Resistance

Avibactam is a first-in-class non–β-lactam β-lactamase inhibitor. It can reinstate the in vitro activity of ceftazidime against Ambler class A, C, and some of D [44]. It was approved by the European Medicines Agency (EMA) for the treatment of patients with cUTI including pyelonephritis, thanks to the RECAPTURE Phase 3 study, which assessed the efficacy and safety of ceftazidime–avibactam [45]. We did not find studies assessing the effectiveness of this treatment against KPCp in UTI. However, we found studies dealing with CAZ-AVI in the context of bacteremia or severe CRE infections that we can extrapolate to the treatment of UTIs with KPCp.

##### Clinical Efficacy, Safety and Tolerability

A multicentric study showed the outcomes of Ceftazidime–Avibactam in patients with Carbapenem-Resistant enterobacterales Infections. Most patients had bacteremia, 17% (8/60) had infections at more than one site, with 28% being primary urinary tract infection, and *Klebsiella pneumoniae* was involved in 83% of the cases.

Fifty-one percent (18/35) of the patients who received Ceftazidime–Avibactam had microbiologic cure, 63% (22/35) had clinical success, and 34% (12/35) died in the hospital [46].

Moreover, another multicenter study presented the interest of CAZ-AVI in salvage therapy in the largest sample to date of patients with infections caused by carbapenem-resistant Gram-negative bacteria, 97% of which included KPC *Klebsiella pneumoniae* (KPC-Kp), while 75.4% were bacteremia and 4.3% were UTI alone. Patients received CAZ-AVI therapy for a median duration of 14 days, and the overall 30-day mortality rate was 34.1% (47/138). The highest rate (36.5% [38/104]) was found in patients with bacteremic KPC-Kp infections, and the lowest (16.7% [1/6]) in those with UTI [47].

A study including 133 patients (46% bacteriemia, 14% UTI) presented evidence for superiority of CAZ-AVI over colistin in the early treatment of KPCp infections. The use of CAZ-AVI was associated with improved clinical outcomes (64%), highlighting reduced all-cause hospital mortality rate (8% vs. 33%), and improved benefit-risk outcomes [48].

The mortality rate did not differ between patients undergoing CAZ-AVI monotherapy compared to CAZ combination therapy for the treatment of CRE infections. This outcome might be able to improve antibiotic treatment, in view of decreasing the use of combination treatments [49,50].

REPRISE, a randomized phase 3 study, comparing ceftazidime-avibactam to the best available therapy in patients with CRE and Pseudomonas aeruginosa cUTI, found adverse events in 28% of patients with CAZ-AVI versus 35% of patients with BAT (nausea 3%, vomiting 3%, diarrhea 2%, fever 3%, and abdominal pain 2% were the first events described). No serious AE or death, were considered, as related to the study drug [51].

An Italian analysis revealed that an empirical treatment with CAZ-AVI followed by colistin and high-dose of carbapenem compared with imipenem followed by colistin and high-dose carbapenem induced a cost of €1015 per patient but offered improved health outcomes in clinical cure (97.65% vs. 91.08%; ∆ = 6.57%), shorter hospital stays (10.65 vs. 12.55 days; ∆ = 1.90 days), and better quality of life (QALYs) (4.190 vs. 4.063; ∆ = 0.126). It randomized 5000 cUTI Italian hospitalized patients in two cohorts: one receiving empirical treatment with CAZ-AVI and the other receiving imipenem. Clinical failure was declared when empirical treatment was switched to the next treatment line if microbiological results revealed that at least one of the pathogens was resistant, if there was no response at the end of the treatment, or if infection recurrence occurred. Every cost was identified (medical costs, management of the recurrence or adverse events).

Empirical treatment resistance would be likely to induce a 10% increase in daily hospitalization cost because of added healthcare resources, a 20% increase in mortality, and a 10% reduction in the success rate of subsequent treatment.

The increasing cost-effectiveness ratio is €8039/QALY, which is under the willingness-to-pay threshold of €30 000/QALY in Italy. These results suggest that CAZ-AVI might be a cost-effective treatment, compared to imipenem association for cUTI [52].

Unfortunately, the latest discoveries indicate that widespread use of CAZ-AVI induces a transformation in the epidemiology of carbapenemases from KPC to metallo-ß-lactamases, particularly in cases of renal dysfunction and/or high inoculum [53].

##### Posology

The suggested dosage of CAZ-AVI is 2 g/0.5 g in 2 h infusions, administered every 8 h in patients 18 years of age and older with estimated glomerular filtration rate (eGFR) superior to 50 mL/min/1.73 m^2^, between 5 to 10 days.

The suggested dose in renally impaired patients with eGFR 31–50 mL/min/1.73 m^2^ is 1 g/0.25 g, for 16–30 mL/min/1.73 m^2^ is 0.75 g/0.1875 g every 12 h, for 6–15 mL/min/1.73 m^2^ is 0.75 g/0.1875 g every 24 h, and for less than 6 mL/min/1.73 m^2^ is 0.75 g/0.1875 g every 48 h [54].

##### PK/PD

The urinary excretion of CEFTAZIDIME-AVIBACTAM is excellent and reaches a targeted MIC of 8 mg/L in 94.9% to even 99.6% in cases of adjusted dosage for renal impairment. Nevertheless, no specific data is available for prostatic diffusion [55].

#### 3.2.4. Imipenem-Cilastatin-Relebactam (Recarbrio^®^)

##### Antimicrobial Spectrum of Activity and Resistance

Relebactam is another non–β-lactam that inhibits class A carbapenemases and class C cephalosporinases [56]. In association with carbapenem imipenem/cilastatin (IMI/REL), it can restore imipenem activity against Imipenem-non-susceptible Gram-negative pathogens as KPC-producing enterobacterales [57]. For KPCp non-susceptible to imipenem, the relebactam reduced the MIC of at least 16-fold, decreasing from 8 to 0.5 μg/mL. The other carbapenemases (OXA-48, VIM or GES-20) are less susceptible to this antibiotic [58]. The European Medicines Agency recognized imipenem-cilastatin-relebactam on the 13th of February 2020 in “the treatment of infections due to aerobic Gram-negative organisms in adults with limited treatment options”.

##### Clinical Efficacy, Safety and Tolerability

RESTORE-IMI 1 was a phase III, randomized, double-blind study, conducted in 35 hospitals, which found that IMI/REL seemed to be a better treatment than colistin + IMI for carbapenem-non-susceptible infections, with similar efficacy but significantly less nephrotoxicity and other adverse events [59].

In this phase III dose-ranging study comparing efficacy and safety of imipenem/cilastatin plus relebactam with imipenem/cilastatin alone in patients with complicated urinary tract infections, the most common adverse events found with IMI/REL 250 mg were headache (7.1%), diarrhea (5.1%), nausea (4.0%), and hypertension (3%) [60].

However, it bears mentioning that IMI/REL has not been studied in cases of severe urosepsis or suspected prostatitis which, as we know, represent a specific tissue. Nevertheless, expert opinion recommends IMI/REL in cUTI when the therapeutic arsenal is restricted [61].

##### Posology

The suggested dosage of IMI/REL in cUTI is 2500 mg/500 mg/250 mg (imipenem/cilastatin/relebactam) in 30 min infusions, administered every 6 h in patients 18 years of age and older with an estimated glomerular filtration rate (eGFR) superior to 90 mL/min/1.73 m^2^, for a duration of 5 to 10 days.

The suggested doses in renally impaired patients with eGFR of 90–60 mL/min/1.73 m^2^ are 400/400/200 g, 300/300/150 g for 60–30 mL/min/1.73 m^2^, 200/200/100 for 30–15 mL/min/1.73 m^2^, and 200/200/100 for less than 15 mL/min/1.73 m^2^ with hemodialysis [62].

##### PK/PD

Urinary excretion of Relebactam is excellent, ranging from 94.7% to 100% over a 24-h period following single-dose administration. Renal clearance is similar when relebactam is administered with or without imipenem-cilastatin. No specific data are available for prostatic diffusion [63].

#### 3.2.5. Plazomicin (Zemdri^®^)

Plazomicin is a novel semisynthetic aminoglycoside of interest against MDR Gram-negatives, including carbapenemase-producers. It was approved by the Food and Drug Administration (FDA) in June 2018 for the treatment of cUTI [64]. A trial comparing plazomicin versus colistin, in association with meropenem or tigecycline (CARE trial) revealed an overall reduction in mortality (24% vs. 50%) [65]. Unfortunately it has not been accepted in Europe in the restricted indication for which it was supposed to be used, due to a lack of economic return compared to the cost of production [66].

#### 3.2.6. Eravacycline (Xerava^®^)

Eravacycline is a fluorocycline with a structure similar to tigecycline [67].

It has been approved by FDA in treatment of intra-abdominal infections and presents favorable in-vitro effects against CRE. Nevertheless, there is a lack of clinical trials to confirm its effectiveness in Europe.

The suggested dosage of Eravacycline is 1 mg/kg in 2 h infusions, administered every 12 h in patients 18 years of age and older, during 4 to 14 days. No dose adjustment for renal or liver function is required [68].

**Table 1:** Characteristics of the main studies included.

	Study, Year Published	Study Design	Study Duration	Study Site	Study Population			No. of Patients (ITT Population)		Dose Regimen	
					Total	UTI	%KPCp	Studied drug	Comparator	Studied drug	Comparator
CEFIDEROCOL: FETROJA^®^	Portsmouth et al., 2018 [29]	Phase 2, Double-blind, non-inferiority trial	2015–2016	65 hospitals in 15 countries	Adults with Gram-negative cUTI	100%	30%	300	148	1-h infusion of cefiderocol (2 g) every 8 h for 7–14 days	1-h infusion of imipenem/cilastatin (1 g each) every 8 h for 7–14 days
	Bassetti et al., 2021 [26] (CREDIBLE-CR trial)	Phase 3, Randomised, open-label, pathogen-focused, descriptive trial	2016–2019	95 hospitals in 16 countries	Adults with NP, BSI or sepsis, or cUTI and a CR-Gram-negative pathogen	24%	26%	101	49	3-h infusion of cefiderocol (2 g) every 8 h for 7–14 days	Best available therapy for 7–14 days
MEROPENEM/ VABORBACTAM: VABOREM^®^	Kaye et al., 2018 [37] (TANGO I CR-trial)	Phase 3, multicenter, multinational, randomised clinical trial, double blind trial	2014–2016	60 hospitals in 17 countries	Adults with complicated UTI, stratified by infection type and geographic region	100%	11%	274	276	Meropenem-vaborbactam (2 g/2 g over 3 h	Piperacillin-tazobactam (4 g/0.5 g over 30 min; every 8 h
	Wunderink et al., 2018 [38] (TANGO II CR-trial)	Phase 3, multinational, open-label, randomized controlled trial	2014–2017	27 hospitals in 8 countries	Adults with infections due to confirmed/suspected CRE	16%	87%	32	15	Meropenem–vaborbactam (2 g/2 g over 3 h, q8h for 7–14 days)	BAT (mono/combination therapy with polymyxins, carbapenems, aminoglycosides, tigecycline; or ceftazidime–avibactam alone)
CEFTAZIDIME-AVIBACTAM: ZAVICEFTA^®^	King et al., 2017 [46]	Multicenter, retrospective review	2015–2016	9 health systems in the United States	Adults who received at least 24 h of ceftazidime–avibactam therapy for CRE infection	28%	83%	29	21	Dosis of ceftazidime–avibactam was determined by providers at each site based on manufacturer’s-recommended dosing	Concomitant therapy and prior therapy for CRE infections were recorded
	Carmeli et al., 2016 [51] (REPRISE)	Phase 3; international, randomised, open-label trial	2013–2014	Hospitals across 16 countries worldwide	Adults with cUTI or cIAI caused by ceftazidime-resistant Enterobacteriaceae or *Pseudomonas aeruginosa*.	92%	38%	165	168	Combination of 2000 mg ceftazidime plus 500 mg avibactam, administered via a 2-h intravenous infusion every 8 h	BAT
IMIPENEM-CILASTATIN-RELEBACTAM: RECARBRIO^®^	Motsch et al., 2020 [59] (RESTORE-IMI 1)	Phase 3, Multicenter, Randomized, controlled Double-blind trial	2015–2017	35 hospitals in 17 countries	Adults hospitalized, and requiring intravenous antibacterial treatment for hospital-acquired pneumonia /ventilator-associated pneumonia, cUTIs, or cIAIs caused by imipenem-nonsusceptible, imipenem/relebactam-susceptible, and colistin-susceptible pathogens and lacking clinical improvement on any prior therapy.	51%	13%	21	10	Intravenous IMI/REL (500 mg/250 mg every 6 h) plus colistimethate sodium placebo	Intravenous IMI/REL (500 mg/250 mg every 6 h) plus intravenous colistimethate sodium (loading dose to achieve 300 mg colistin base activity, followed by maintenance doses up to 150 mg colistin base activity, every 12 h)

**Table 2:** Summaries of main characteristics of these new antibiotic drugs and their efficacy on KPCp UTIs.

Antibiotics and Classes	Action Spectre, PK/PD Data	Posology	AEs	Notes
CEFIDEROCOL (FETCROJA^®^), a siderophore cephalosporin	-Amber Class A, B, C and D enterobacteriales. Not active against gram-positive aerobic bacteria and anaerobic bacteria -The urinary excretion is ranged from 61.5% to 68.4% unchanged antibiotic product regardless of the dosage	2 g administered every 8 h with an eGFR superior to 90 mL/min/1.73 m^2^ between five to ten days	Diarrhea (19%), fever (14%) and vomiting (13%)	it is the only molecule that has activity on all carbapenemases. It brings a benefit in terms of mechanism of action and diversity of sites of action. To use exclusively as a last resort for reasons of preservation.
MEROPENEM/ VABORBACTAM (VABOREM^®^), a Carbapenem + non-β-lactam, serine beta-lactamase inhibitor	Aerobic and anaerobic Gram-positive and negative, ESBL and AmpC producing enterobacteriae, Amber Class-A enterobacterial -Urinary excretion of Meropenem and Vaborbactam ranged from 40 to 60% and 75 to 95%respectively. No specific data are available for prostatic diffusion	Meropenem 2 g and vaborbactam 2 g administered every 8 h with an eGFR superior to 50 mmL/min/1.73 m^2^, between five to ten days	Headache (3.8–21.6%), infusion site phlebitis (42–62.2%), nausea (19.5%), and diarrhea (14.6%)	M/V shows a better safety profile compared to BAT and presents fewer adverse events
CEFTAZIDIME-AVIBACTAM (Zavicefta^®^), a third generation cephalosporine + non–β-lactam β-lactamase inhibitor	-Ambler class A, C and some of D enterobacteriales -Urinary excretion is excellent and joins a targeted MIC of 8 mg/L in 94.9% to 99.6% even in case of adjusted dosage for renal impairment.	2 g/0.5 g in 2 h infusions, administered every 8 h with an eGFR superior to 50 mL/min/1.73 m^2^, between 5 to 10 days.	Nausea 3%, vomiting 3%, diarrhea 2%, Pyrexia 3%, abdominal pain 2%	Reduces all-cause hospital mortality rate Delay in starting CAZ-AVI may not impact the survival and is a good option for salvage. Latest discoveries indicate that widespread use of CAZ-AVI produced a transformation in epidemiology of carbapenemases from KPCp to metallo-b-lactamases.
IMIPENEM-CILASTATIN-RELEBACTAM (RECARBRIO^®^), a Carbapenem + dehydropeptidase inhibitors + non–β-lactam β-lactamase inhibitor	-Amber class A carbapenemases and class C cephalosporinases -Urinary excretion of Relebactam is excellent and ranged from 94.7% to 100% over a 24-h period following single-dose administration. Renal clearance is similar when relebactam is administered with and without imipenem-cilastatin	2500 mg/500 mg/250 mg (imipenem/cilastatin/relebactam) in 30 min infusions, administered every 6 h with an eGFR superior to 90 mL/min/1.73 m^2^	Less nephrotoxicity, headache (7.1%), diarrhea (5.1%), nausea (4.0%) and hypertension (3%)	IMI/REL have not been studied in case of severe urosepsis or suspected prostatitis Nevertheless, expert opinion recommends IMI/REL in cUTI when the therapeutic arsenal is restricted

## 4. Discussion

Imipenem-cilastatin-relebactam, meropenem-vaborbactam, cefiderocol and ceftazidime–avibactam represent a major step forward in management of adult patients with UTI caused by carbapenemase-producing enterobacterales. However, there are no guidelines to choose the most appropriate among these antibiotics, according to the characteristics of each and to the clinical situation. Indeed, “UTI” represents a highly heterogeneous group of infections (cystitis, prostatitis, pyelonephritis, etc.) and further studies will be necessary to determine the best antibiotic treatment option for each.

We tried to retain the advantages of each of the above-mentioned molecules to optimally guide KPCp UTI management.

### 4.1. Cefiderocol (Fetroja^®^)

Very few trials have been carried out on carbapenem-resistant bacteria and none have reached the scale of CREDIBLE, which includes over 100 patients, more than half of whom are severe, which is rare in the records on this type of molecule but representative of the reality.

It differs from the other antibiotics by its mechanism of action, which is the only one of its kind, with penetration through the iron transport system rather than through the porins, facilitating activity on impermeable bacteria [23,24,25]. Gram Negative Bacteria (GNB) often have multiple resistance mechanisms that are not only enzymatic, but also porin impermeability. This is important because cefiderocol can counteract both mechanisms [24,25].

Indeed, it is the only molecule with activity on all carbapenemases, whereas the others are very specific, with restricted targets, either KPC or OXA-48. The efficacy of this antibiotic in monotherapy on metallo-beta-lactamases is exceptional.

The other, less innovative molecules are combinations of old beta-lactam antibiotics with new inhibitors, whereas by its mechanism of action this molecule brings a real benefit, not only in terms of mechanism of action and diversity of sites of action, but also due to a much wider spectrum than the other molecules covered up until now.

Given the characteristics of the product and the need for reasons of preservation to restrict the use to last resort, the decision to initiate treatment with cefiderocol should only be made following a complete antibiogram, and after a discussion with multidisciplinary antibiotic referents, with systematic re-evaluation 48 h after treatment initiation. It bears mentioning that cefiderocol is not active against Gram-positive aerobic and anaerobic bacteria. Other antibiotics should be used if these pathogens are known or suspected to be involved in the infection.

### 4.2. Meropenem/Vaborbactam (Vaborem^®^)

The efficacy of combined meropenem/vaborbactam was assessed in the TANGO II study in adult patients with confirmed or suspected carbapenem-resistant enterobacterale (CRE) infections (38). These results should be interpreted with caution considering the small number of patients (32 patients treated with VABOREM vs. 15 patients treated with BAT) and the study design (open-label, descriptive analysis). All in all, a more favorable response in terms of clinical cure, microbiological eradication and all-cause mortality was observed in the meropenem+vaborbactam group than in the control group. Its efficacy has been demonstrated in complicated urinary tract infections of low to moderate severity, including pyelonephritis due to BGN, but data on severe forms are limited. From our perspective, M/V should be considered as the gold standard for the treatment of infections due to UTI.

### 4.3. Ceftazidime-Avibactam (Zavicefta^®^)

CAZ-AVI was first approved in the United States in February 2015 and has since been approved in more than 47 countries. Cumulative exposure since the beginning of its marketing has been estimated at 43,633 patients, making it one of the most well-documented compounds of its kind. Hypersensitivity reaction is the most widely reported side effect and is mainly due to the presence of ceftazidime. Before treatment is initiated, it is necessary to determine patient’s history of hypersensitivity reactions to ceftazidime, other cephalosporins, or any other β-lactam antibiotic.

The combination of ceftazidime and avibactam has been shown to be effective in treatment of urinary tract infections (including pyelonephritis) [46,47,48,49,50,51]. Data on severe forms and/or forms caused by multi-resistant bacteria are limited, especially regarding beta-lactamase-producing enterobacterales. Nevertheless, bacteriological data and clinical experience reported in observational studies suggest that CAZ-AVI should in the absence of acquired resistance mechanisms be effective in infections due to certain carbapenemases (such as KPC). However, these mechanisms seem to be more and more frequent as we have observed a transformation in the epidemiology of KPC towards MBL bacteria, which subsequently present cross-resistance to CAZ-AVI, meropenem/vaborbactam and imipenem/relebactam [53].

The major advantage of CAZ-AVI is its marketing authorization for children aged 3 months to 18 years with regard to infections caused by enterobacterales sensitive to the ceftazidime/avibactam combination and for whom the use of other beta-lactams and carbapenems (meropenem or imipenem-cilastatin) is not an option in the event of resistance, particularly on account of the production of KPC-type carbapénémases.

### 4.4. Imipenem-Cilastatin-Relebactam (Recarbrio^®^)

The main study on imipenem-relebactam in KPCp UTI (RESTORE-IMI-1) covers a very small population [59]; while 50 patients were screened, but only 31 patients, 21 in the imipenem-relebactam group and 10 in the imipenem-colistin group, were considered in the microbiologically modified ITT analysis. When we look more closely, among the 16 patients with urinary tract infection, only 16% were *K. Pneumoniae*. Concerning resistance mechanisms, there were only six carbapenemase-producing enterobacterales, five KPC and one OXA-48. In terms of tolerability, there was no significant differences: 71% in the IMI-REL group versus 81% in the IMI-COL group, but the numbers were not relevant in terms of power. This is a case report-based study, conducted on a small number of relevant patients. IMI/REL has not been studied in cases of severe urosepsis or suspected prostatitis. There is no strong clinical support to appraise its results, which are based in vitro microbiology data and PK/PD data on imipenem-relebactam. While compared to ceftazidime–avibactam and meropenem-vaborbactam, imipenem-relebactam represents a potential alternative therapy in KPCp infections, we are mainly relying on microbiological data, and not sufficiently on clinical data.

### 4.5. Perspectives

Due to the lack of scientific data, it is difficult to recommend a single and simple way to treat KPCp-UTI. Even if these infections are extremely difficult to treat, they rarely occur, and it is difficult to put together a large cohort with homogeneous patients. A strong partnership between countries, teams and hospitals is probably an indispensable prerequisite to the creation of large cohorts of KPCp-UTI, which could lead to high level of evidence studies on this topic.

In addition, antibiotic drugs represent only one perspective to treat these infections. For example, essential oils could represent an interesting alternative or potentiator of antibiotic drugs [69,70].

Finally, the best treatment of KPCp-UTI is probably prevention. It has been demonstrated that low or better prescription of antibiotic drugs reduces the level of antibiotic resistance [71]. In light of the study by Klein et al. [72], a global policy regarding antibiotic stewardship should be established and published, the objective being to achieve better prevention of antibiotic consumption throughout the world.

## 5. Conclusions

KPCp-UTIs are a real challenge for physicians. While Cefiderocol, Meropenem-vaborbactam, Ceftazidim-avibactam and Imipenem-relebactam represent a major step forward in treatment of these UTIs, unfortunately, no guidelines are currently available in view of choosing the most appropriate treatment in each specific case. Due to the absence of guidelines, both MIC and molecular biology testing should always be initially performed t in case of suspicion of multidrug resistant infection, and especially in case of KPCp UTI, the objective being to provide clinicians with several possibilities of treatment for complicated infections in frequently high-morbidity patients.

## References

[1] Cassini A., Högberg L.D., Plachouras D., Quattrocchi A., Hoxha A., Simonsen G.S., Colomb-Cotinat M., Kretzschmar M.E., Devleesschauwer B., Cecchini M. (2019). Attributable deaths and disability-adjusted life-years caused by infections with antibiotic-resistant bacteria in the EU and the European Economic Area in 2015: A population-level modelling analysis. Lancet Infect. Dis..

[2] Theuretzbacher U. (2017). Global antimicrobial resistance in Gram-negative pathogens and clinical need. Curr. Opin. Microbiol..

[3] Tacconelli E., Carrara E., Savoldi A., Harbarth S., Mendelson M., Monnet D.L., Pulcini C., Kahlmeter G., Kluytmans J., Carmeli Y. (2018). Discovery, research, and development of new antibiotics: The WHO priority list of antibiotic-resistant bacteria and tuberculosis. Lancet Infect. Dis..

[4] World Health Organization (2014). Antimicrobial Resistance: Global Report on Surveillance.

[5] Zilberberg M.D., Shorr A.F. (2013). Secular Trends in Gram-Negative Resistance among Urinary Tract Infection Hospitalizations in the United States, 2000–2009. Infect. Control. Hosp. Epidemiol..

[6] Nordmann P., Naas T., Poirel L. (2011). Global spread of Carbapenemase-producing *Enterobacteriaceae*. Emerg. Infect. Dis..

[7] Rodriguez-Gómez J., Pérez-Nadales E., Gutiérrez-Gutiérrez B., Machuca I., Martinez-Martinez L., Rivera F., Cano A., Castón J.J., Robles J.C., de la Fuente C. (2019). Prognosis of urinary tract infection caused by KPC-producing *Klebsiella pneumoniae*: The impact of inappropriate empirical treatment. J. Infect..

[8] Nordmann P., Poirel L. (2019). Epidemiology and Diagnostics of Carbapenem Resistance in Gram-negative Bacteria. Clin. Infect. Dis..

[9] Bush K., Bradford P.A. (2020). Epidemiology of β-Lactamase-Producing Pathogens. Clin. Microbiol. Rev..

[10] Alexander B.T., Marschall J., Tibbetts R.J., Neuner E.A., Dunne W.M., Ritchie D.J. (2012). Treatment and clinical outcomes of urinary tract infections caused by KPC-producing *Enterobacteriaceae* in a retrospective cohort. Clin. Ther..

[11] Page M.J., McKenzie J.E., Bossuyt P.M., Boutron I., Hoffmann T.C., Mulrow C.D., Shamseer L., Tetzlaff J.M., Akl E.A., Brennan S.E. (2021). The PRISMA 2020 statement: An updated guideline for reporting systematic reviews. BMJ.

[12] Meletis G. (2016). Carbapenem resistance: Overview of the problem and future perspectives. Ther. Adv. Infect. Dis..

[13] Logan L.K., Weinstein R.A. (2017). The Epidemiology of Carbapenem-Resistant *Enterobacteriaceae*: The Impact and Evolution of a Global Menace. J. Infect. Dis..

[14] Bush K., Jacoby G.A. (2010). Updated Functional Classification of β-Lactamases. Antimicrob. Agents Chemother..

[15] Urmi U.L., Nahar S., Rana M., Sultana F., Jahan N., Hossain B., Alam M.S., Mosaddek A.S.M., McKimm J., Rahman N.A.A. (2020). Genotypic to Phenotypic Resistance Discrepancies Identified Involving β-Lactamase Genes, *blaKPC*, *blaIMP*, *blaNDM-1*, and *blaVIM* in Uropathogenic *Klebsiella pneumoniae*. Infect. Drug Resist..

[16] Patel G., Bonomo R.A. (2013). Stormy waters ahead: Global emergence of carbapenemases. Front. Microbiol..

[17] Queenan A.M., Bush K. (2007). Carbapenemases: The versatile beta-lactamases. Clin. Microbiol. Rev..

[18] Nordmann P., Cuzon G., Naas T. (2009). The real threat of *Klebsiella pneumoniae* carbapenemase-producing bacteria. Lancet Infect. Dis..

[19] Van Duin D., Doi Y. (2017). The global epidemiology of carbapenemase-producing *Enterobacteriaceae*. Virulence.

[20] Pang F., Jia X.-Q., Zhao Q.-G., Zhang Y. (2018). Factors associated to prevalence and treatment of carbapenem-resistant *Enterobacteriaceae* infections: A seven years retrospective study in three tertiary care hospitals. Ann. Clin. Microbiol. Antimicrob..

[21] Van Duin D., Kaye K.S., Neuner E.A., Bonomo R.A. (2013). Carbapenem-resistant *Enterobacteriaceae*: A review of treatment and outcomes. Diagn. Microbiol. Infect. Dis..

[22] Neuner E.A., Yeh J.-Y., Hall G.S., Sekeres J., Endimiani A., Bonomo R.A., Shrestha N.K., Fraser T.G., van Duin D. (2011). Treatment and Outcomes in Carbapenem-resistant *Klebsiella pneumoniae* Bloodstream Infections. Diagn. Microbiol. Infect. Dis..

[23] Hackel M.A., Tsuji M., Yamano Y., Echols R., Karlowsky J.A., Sahm D.F. (2017). In Vitro Activity of the Siderophore Cephalosporin, Cefiderocol, against a Recent Collection of Clinically Relevant Gram-Negative Bacilli from North America and Europe, Including Carbapenem-Nonsusceptible Isolates (SIDERO-WT-2014 Study). Antimicrob. Agents Chemother..

[24] Domalaon R., Idowu T., Zhanel G.G., Schweizer F. (2018). Antibiotic Hybrids: The Next Generation of Agents and Adjuvants against Gram-Negative Pathogens?. Clin. Microbiol. Rev..

[25] Ito-Horiyama T., Ishii Y., Ito A., Sato T., Nakamura R., Fukuhara N., Tsuji M., Yamano Y., Yamaguchi K., Tateda K. (2016). Stability of Novel Siderophore Cephalosporin S-649266 against Clinically Relevant Carbapenemases. Antimicrob. Agents Chemother..

[26] Bassetti M., Echols R., Matsunaga Y., Ariyasu M., Doi Y., Ferrer R., Lodise T.P., Naas T., Niki Y., Paterson D.L. (2021). Efficacy and safety of cefiderocol or best available therapy for the treatment of serious infections caused by carbapenem-resistant Gram-negative bacteria (CREDIBLE-CR): A randomised, open-label, multicentre, pathogen-focused, descriptive, phase 3 trial. Lancet Infect. Dis..

[27] (2021). Cefiderocol versus high-dose, extended-infusion meropenem for the treatment of Gram-negative nosocomial pneumonia (APEKS-NP): A randomised, double-blind, phase 3, non-inferiority trial. Lancet Infect. Dis..

[28] Hsueh S.-C., Chao C.-M., Wang C.-Y., Lai C.-C., Chen C.-H. (2021). Clinical efficacy and safety of cefiderocol in the treatment of acute bacterial infections: A systematic review and meta-analysis of randomised controlled trials. J. Glob. Antimicrob. Resist..

[29] Portsmouth S., van Veenhuyzen D., Echols R., Machida M., Ferreira J.C.A., Ariyasu M., Tenke P., Den Nagata T. (2018). Cefiderocol versus imipenem-cilastatin for the treatment of complicated urinary tract infections caused by Gram-negative uropathogens: A phase 2, randomised, double-blind, non-inferiority trial. Lancet Infect. Dis..

[30] Katsube T., Echols R., Arjona Ferreira J.C., Krenz H.K., Berg J.K., Galloway C. (2017). Cefiderocol, a Siderophore Cephalosporin for Gram-Negative Bacterial Infections: Pharmacokinetics and Safety in Subjects With Renal Impairment. J. Clin. Pharmacol..

[31] Fetroja (Cefiderocol) Dosing, Indications, Interactions, Adverse Effects, and More. https://reference.medscape.com/drug/fetroja-cefiderocol-4000008.

[32] Saisho Y., Katsube T., White S., Fukase H., Shimada J. (2018). Pharmacokinetics, Safety, and Tolerability of Cefiderocol, a Novel Siderophore Cephalosporin for Gram-Negative Bacteria, in Healthy Subjects. Antimicrob. Agents Chemother..

[33] FDA (2020). Commissioner O of the FDA Approves New Antibacterial Drug. https://www.fda.gov/news-events/press-announcements/fda-approves-new-antibacterial-drug.

[34] Baldwin C.M., Lyseng-Williamson K.A., Keam S.J. (2008). Meropenem: A review of its use in the treatment of serious bacterial infections. Drugs.

[35] Rechenchoski D.Z., Dambrozio A.M.L., Vivan A.C.P., Schuroff P.A., Burgos T.d.N., Pelisson M., Perugini M.R.E., Vespero E.C. (2017). Antimicrobial activity evaluation and comparison of methods of susceptibility for *Klebsiella pneumoniae* carbapenemase (KPC)-producing *Enterobacter* spp. isolates. Braz. J. Microbiol..

[36] Lomovskaya O., Sun D., Rubio-Aparicio D., Nelson K., Tsivkovski R., Griffith D.C., Dudley M.N. (2017). Vaborbactam: Spectrum of Beta-Lactamase Inhibition and Impact of Resistance Mechanisms on Activity in *Enterobacteriaceae*. Antimicrob. Agents Chemother..

[37] Kaye K.S., Bhowmick T., Metallidis S., Bleasdale S.C., Sagan O.S., Stus V., Vazquez J., Zaitsev V., Bidair M., Chorvat E. (2018). Effect of Meropenem-Vaborbactam vs Piperacillin-Tazobactam on Clinical Cure or Improvement and Microbial Eradication in Complicated Urinary Tract Infection: The TANGO I Randomized Clinical Trial. JAMA.

[38] Wunderink R.G., Giamarellos-Bourboulis E.J., Rahav G., Mathers A.J., Bassetti M., Vazquez J., Cornely O.A., Solomkin J., Bhowmick T., Bishara J. (2018). Effect and Safety of Meropenem-Vaborbactam versus Best-Available Therapy in Patients with Carbapenem-Resistant *Enterobacteriaceae* Infections: The TANGO II Randomized Clinical Trial. Infect. Dis. Ther..

[39] Patel T.S., Pogue J.M., Mills J.P., Kaye K.S. (2018). Meropenem-vaborbactam: A new weapon in the war against infections due to resistant Gram-negative bacteria. Future Microbiol..

[40] Wu G., Cheon E. (2018). Meropenem-vaborbactam for the treatment of complicated urinary tract infections including acute pyelonephritis. Expert Opin. Pharmacother..

[41] Burgos R.M., Biagi M.J., Rodvold K.A., Danziger L.H. (2018). Pharmacokinetic evaluation of meropenem and vaborbactam for the treatment of urinary tract infection. Expert Opin. Drug Metab. Toxicol..

[42] Vabomere (Meropenem/Vaborbactam) Dosing, Indications, Interactions, Adverse Effects, and More. https://reference.medscape.com/drug/vabomere-meropenem-vaborbactam-1000130.

[43] Trang M., Griffith D.C., Bhavnani S.M., Loutit J.S., Dudley M.N., Ambrose P.G., Rubino C.M. (2021). Population Pharmacokinetics of Meropenem and Vaborbactam Based on Data from Noninfected Subjects and Infected Patients. Antimicrob. Agents Chemother..

[44] Lahiri S.D., Mangani S., Durand-Reville T., Benvenuti M., De Luca F., Sanyal G., Docquier J.D. (2013). Structural Insight into Potent Broad-Spectrum Inhibition with Reversible Recyclization Mechanism: Avibactam in Complex with CTX-M-15 and *Pseudomonas aeruginosa* AmpC β-Lactamases. Antimicrob. Agents Chemother..

[45] Wagenlehner F.M., Sobel J.D., Newell P., Armstrong J., Huang X., Stone G.G., Yates K., Gasink L.B. (2016). Ceftazidime-avibactam Versus Doripenem for the Treatment of Complicated Urinary Tract Infections, Including Acute Pyelonephritis: RECAPTURE, a Phase 3 Randomized Trial Program. Clin. Infect. Dis..

[46] King M., Heil E., Kuriakose S., Bias T., Huang V., El-Beyrouty C., McCoy D., Hiles J., Richards L., Gardner J. (2017). Multicenter Study of Outcomes with Ceftazidime-Avibactam in Patients with Carbapenem-Resistant *Enterobacteriaceae* Infections. Antimicrob. Agents Chemother..

[47] Tumbarello M., Trecarichi E.M., Corona A., De Rosa F.G., Bassetti M., Mussini C., Menichetti F., Viscoli C., Campoli C., Venditti M. (2019). Efficacy of Ceftazidime-Avibactam Salvage Therapy in Patients With Infections Caused by *Klebsiella pneumoniae* Carbapenemase–producing *K. pneumoniae*. Clin. Infect. Dis..

[48] Van Duin D., Lok J.J., Earley M., Cober E., Richter S.S., Perez F., Salata R.A., Kalayjian R.C., Watkins R.R., Doi Y. (2018). Colistin Versus Ceftazidime-Avibactam in the Treatment of Infections Due to Carbapenem-Resistant Enterobacteriaceae. Clin. Infect. Dis..

[49] Leone S., Cascella M., Pezone I., Fiore M. (2019). New antibiotics for the treatment of serious infections in intensive care unit patients. Curr. Med. Res. Opin..

[50] Fiore M., Alfieri A., Di Franco S., Pace M.C., Simeon V., Ingoglia G., Cortegiani A. (2020). Ceftazidime-Avibactam Combination Therapy Compared to Ceftazidime-Avibactam Monotherapy for the Treatment of Severe Infections Due to Carbapenem-Resistant Pathogens: A Systematic Review and Network Meta-Analysis. Antibiotics.

[51] Carmeli Y., Armstrong J., Laud P.J., Newell P., Stone G., Wardman A., Gasink L.B. (2016). Ceftazidime-avibactam or best available therapy in patients with ceftazidime-resistant *Enterobacteriaceae* and *Pseudomonas aeruginosa* complicated urinary tract infections or complicated intra-abdominal infections (REPRISE): A randomised, pathogen-directed, phase 3 study. Lancet Infect. Dis..

[52] Kongnakorn T., Wagenlehner F., Falcone M., Tichy E., Di Virgilio R., Baillon-Plot N., Charbonneau C. (2019). Cost-effectiveness analysis of ceftazidime/avibactam compared to imipenem as empirical treatment for complicated urinary tract infections. Int. J. Antimicrob. Agents.

[53] Papadimitriou-Olivgeris M., Bartzavali C., Lambropoulou A., Solomou A., Tsiata E., Anastassiou E.D., Fligou F., Marangos M., Spiliopoulou I., Christofidou M. (2019). Reversal of carbapenemase-producing *Klebsiella pneumoniae* epidemiology from blaKPC- to blaVIM-harbouring isolates in a Greek ICU after introduction of ceftazidime/avibactam. J. Antimicrob. Chemother..

[54] VIDAL ZAVICEFTA 2 g/0.5 g pdre p sol diluer p perf. https://www.vidal.fr/medicaments/zavicefta-2-g-0-5-g-pdre-p-sol-diluer-p-perf-172057.html.

[55] Das S., Li J., Riccobene T., Carrothers T.J., Newell P., Melnick D., Critchley I.A., Stone G.G., Nichols W.W. (2019). Dose Selection and Validation for Ceftazidime-Avibactam in Adults with Complicated Intra-abdominal Infections, Complicated Urinary Tract Infections, and Nosocomial Pneumonia. Antimicrob. Agents Chemother..

[56] Livermore D.M., Warner M., Mushtaq S. (2013). Activity of MK-7655 combined with imipenem against *Enterobacteriaceae* and *Pseudomonas aeruginosa*. J. Antimicrob. Chemother..

[57] Zhanel G.G., Lawrence C.K., Adam H., Schweizer F., Zelenitsky S., Zhanel M., Lagacé-Wiens P.R., Walkty A., Denisuik A., Golden A. (2018). Imipenem-Relebactam and Meropenem-Vaborbactam: Two Novel Carbapenem-β-Lactamase Inhibitor Combinations. Drugs.

[58] Lob S.H., Hackel M.A., Kazmierczak K.M., Young K., Motyl M.R., Karlowsky J.A., Sahm D.F. (2017). In Vitro Activity of Imipenem-Relebactam against Gram-Negative ESKAPE Pathogens Isolated by Clinical Laboratories in the United States in 2015 (Results from the SMART Global Surveillance Program). Antimicrob. Agents Chemother..

[59] Motsch J., Murta de Oliveira C., Stus V., Köksal I., Lyulko O., Boucher H.W., Kaye K.S., File T.M., Brown M.L., Khan I. (2020). RESTORE-IMI 1: A Multicenter, Randomized, Double-blind Trial Comparing Efficacy and Safety of Imipenem/Relebactam vs Colistin Plus Imipenem in Patients With Imipenem-nonsusceptible Bacterial Infections. Clin. Infect. Dis..

[60] Sims M., Mariyanovski V., McLeroth P., Akers W., Lee Y.-C., Brown M.L., Du J., Pedley A., Kartsonis N.A., Paschke A. (2017). Prospective, randomized, double-blind, Phase 2 dose-ranging study comparing efficacy and safety of imipenem/cilastatin plus relebactam with imipenem/cilastatin alone in patients with complicated urinary tract infections. J. Antimicrob. Chemother..

[61] Kuiper S.G., Leegwater E., Wilms E.B., van Nieuwkoop C. (2020). Evaluating imipenem + cilastatin + relebactam for the treatment of complicated urinary tract infections. Expert Opin. Pharmacother..

[62] VIDAL RECARBRIO: Association Fixe D’imipénem, de Cilastatine et D’un Nouvel Antibiotique, le Rélébactamcited. https://www.vidal.fr/actualites/26443-recarbrio-association-fixe-d-imipenem-de-cilastatine-et-d-un-nouvel-antibiotique-le-relebactam.html.

[63] Rhee E.G., Rizk M.L., Calder N., Nefliu M., Warrington S.J., Schwartz M.S., Mangin E., Boundy K., Bhagunde P., Colon-Gonzalez F. (2018). Pharmacokinetics, Safety, and Tolerability of Single and Multiple Doses of Relebactam, a β-Lactamase Inhibitor, in Combination with Imipenem and Cilastatin in Healthy Participants. Antimicrob. Agents Chemother..

[64] Bilinskaya A., Linder K.E., Kuti J.L. (2020). Plazomicin: An intravenous aminoglycoside antibacterial for the treatment of complicated urinary tract infections. Expert Rev. Anti-Infect. Ther..

[65] McKinnell J.A., Dwyer J.P., Talbot G.H., Connolly L.E., Friedland I., Smith A., Jubb A.M., Serio A.W., Krause K.M., Daikos G.L. (2019). Plazomicin for Infections Caused by Carbapenem-Resistant *Enterobacteriaceae*. N. Engl. J. Med..

[66] Rex J., Outterson K. (2020). Plazomicin EU Marketing Application is Withdrawn: Near Zero Market Value of Newly Approved Antibacterials. AMR.Solutions. https://amr.solutions/2020/07/11/plazomicin-eu-marketing-application-is-withdrawn-near-zero-market-value-of-newly-approved-antibacterials/.

[67] Zhanel G.G., Cheung D., Adam H., Zelenitsky S., Golden A., Schweizer F., Gorityala B., Lagacé-Wiens P.R., Walkty A., Gin A.S. (2016). Review of Eravacycline, a Novel Fluorocycline Antibacterial Agent. Drugs.

[68] Mahieu R., Dubée V. (2020). Nouveaux antibiotiques. Réanimation.

[69] Yang S.-K., Yusoff K., Thomas W., Akseer R., Alhosani M.S., Abushelaibi A., Lai K.S. (2020). Lavender essential oil induces oxidative stress which modifies the bacterial membrane permeability of carbapenemase producing *Klebsiella pneumoniae*. Sci. Rep..

[70] Trong Le N., Viet Ho D., Quoc Doan T., Tuan Le A., Raal A., Usai D., Madeddu S., Marchetti M., Usai M., Rappelli P. (2020). In vitro Antimicrobial Activity of Essential Oil Extracted from Leaves of *Leoheo domatiophorus* Chaowasku, D.T. Ngo and H.T. Le in Vietnam. Plants.

[71] Maugat S., Berger-carbonne A. (2019). Antibiotiques et Résistance Bactérienne: Une Menace Mondiale, Des Conséquences Individuelles.

[72] Klein E.Y., Boeckel T.P.V., Martinez E.M., Pant S., Gandra S., Levin S.A., Goossens H., Laxminarayan R. (2018). Global increase and geographic convergence in antibiotic consumption between 2000 and 2015. Proc. Natl. Acad. Sci. USA.

